# Synthesis and Evaluation of Biological Activity of New Arylphosphoramidates

**DOI:** 10.1155/2018/4567019

**Published:** 2018-08-26

**Authors:** Amal Thebti, M. A. K. Sanhoury, H-I. Ouzari, T. Barhoumi-slimi

**Affiliations:** ^1^LR03ES03 Laboratoire des Microorganismes et Biomolécules Actives, Département de Biologie, Faculté des Sciences de Tunis, Université ElManar, 2092, Tunisia; ^2^LR99ES14 Laboratory of Structural Organic Chemistry, Synthesis and Physicochemical Studies, Department of Chemistry, Faculty of Sciences of Tunis, University of Tunis El Manar, 2092 Tunis, Tunisia; ^3^High Institute of Environmental Science and Technology, University of Carthage, Technopark of Borj-Cedria BP-1003, Hammam-Lif 2050, Tunisia

## Abstract

The synthesis of new substituted arylphosphoramidates is performed in two steps through phosphorylation of the corresponding alcohols followed by aminolysis. The formation of the desired phosphoramidates depends on the subsequent addition of the two alcohols with the amine being added at the last step. The products were obtained in 58–95% yields. They were characterized mainly by multinuclear (^1^H, ^13^C, ^31^P, and ^19^F) NMR and IR spectroscopy. In addition, the antimicrobial and antiacetylcholinesterase activities were evaluated. The results showed acetylcholinesterase activity by some compounds, whilst no significant inhibitory effect against the tested bacterial strains has been recorded.

## 1. Introduction

Organophosphorus compounds are widely used as pesticides and chemical weapon agents because of their inhibitory effect on acetylcholinesterase [1]. The development in the field of medicinal chemistry of these compounds is currently characterized by a more marked orientation towards the synthesis of their derivatives as prodrugs for pharmaceutical purposes [2, 3]. Recent studies have shown that phosphoramidates and phosphates can be used as anticancer agents [4, 5], anti-HIV [6], and against Alzheimer's disease [7]. It was shown [8] that some phosphoramidates are active against strains of* Bacillus subtilis*,* Escherichia coli*,* Staphylococcus aureus*, and* Streptococcus mutans. *It was also shown [9] that they are bacterial enzyme inhibitors, aspartate semialdehyde dehydrogenase (ASA-DH), which is involved in the biosynthesis of the aspartate family of amino acids. The biological activity of these compounds was also shown to depend significantly on the phosphorus atom substituents [10]. Thus, p-nitrophenylphosphoramidate derivatives were proven to be considerably stronger [11] than the methamidophos which is known for its acetylcholinesterase (AChE) inhibition and insecticidal property [12]. Furthermore, it was shown that phosphoramidates could be very useful for studying the mechanism of prophylaxis against poisoning by organophosphates and also reported that the p-nitrophenylphosphoramidates protect the guinea pigs against poisoning by Soman neurotoxic gas [13].

Inspired by these results and in continuation of our research on the complexing properties of phosphorylated compounds [14–17], we have already studied in a previous paper the complexes SnCl_4_.2L by multinuclear NMR at variable temperature of a series of new arylphosphoramidates with the formula (ArO)P(O)(NR_2_)(OR′) [18]. We have found that tuning substituents nondirectly bounded at phosphorus atom as R, R', and Ar groups have affected the donor character of the phosphoryl group towards tin atom. In this paper we describe the synthesis of these arylphosphoramidates and their biological activity tests against bacterial strains and acetylcholinesterase enzyme.

## 2. Materials and Methods

### 2.1. Chemistry

#### 2.1.1. Synthesis of Phosphoramidates

All reactions were performed under nitrogen using anhydrous solvents. The Et_3_N products, POCl_3_, Me_2_NH, Et_2_NH, piperidine, morpholine, p-cresol, nitrophenol, and 2,2,2-trifluoroethanol, are commercial. The liquids are distilled before use and the solvents were dried by conventional methods. The synthesis of CF_3_CH_2_OP(O)Cl_2_ was performed according to the literature [19] and the synthesis of 4-nitrophenyldichlophosphate was also performed according to literature [20]. The proton NMR spectra (TMS) at 300 MHz, ^31^P (H_3_PO_4_ 85%) at 121 MHz, and ^19^F (CFCl_3_) at 282 MHz were recorded on a Bruker AVANCE III-300. HRMS were recorded on Q-Tof 6500 Series.

#### 2.1.2. Synthesis of 4-methylphenyl-2,2,2-trifluoroethylchlorophosphate

In an Erlenmeyer flask surmounted by a funnel under nitrogen, a solution of 2,2,2-trifluoroethyldichlorophosphate (16 mmol) was introduced into 120 mL of anhydrous ether. The mixture of p-cresol (16 mmol) with triethylamine (16 mmol) in 50 mL of anhydrous ether was added dropwise at room temperature. After 12 hours of stirring, the precipitate was removed by filtration and the filtrate concentrated distilled. A pale yellow liquid was obtained, with yield = 93%, E_b0,3mmHg_ = 80°C. RMN* δ*: ^31^P: 0.52; ^1^H**:** 2.35 (s, CH_3_-Ph); 4.5 (m, OCH_2_); 7.5 (m, -Ph-, 4H).

#### 2.1.3. Synthesis of 4-methylphenyl-2,2,2-trifluoroethyl phosphoramidates

A solution of 2,2,2-trifluoroethyldichlorophosphate (15 mmol) was placed in 100 mL of anhydrous ether in a flask equipped with a funnel under a flew of nitrogen, then HNR_2_ (30 mmol) in 20 mL of anhydrous ether is added dropwise. Viscous liquid is obtained without no further purification. The ^31^P NMR spectra show that crude compounds are pure.

**4-Methylphenyl-2,2,2-trifluoroethyl dimethylamidophosphate 3a is as follows: **It is a colorless viscous liquid, yielding 92%; ^1^H NMR *δ*: 2.32 (CH_3_-Ph); 2.76 (d, CH_3_N, 6H, ^3^J_H-P_ = 12 Hz); 4.32 (m, OCH_2_); 7.1 (m, -Ph-, 4H). ^19^F NMR *δ ***: −**75.27 (t, ^3^J_H-F_ = 8.5 Hz). ^13^C NMR *δ ***:** 20.6 (CH_3_Ph); 36.5 (CH_3_N); 62.7 (q, CH_2_CF_3_; J = 37 Hz); 122 (q, CF_3_, J = 276 Hz); (117; 130; 134; 148; C_arom_). ^31^P NMR *δ ***:** 6.15 (9 peaks, ^3^J_P-H_ = 9.7Hz).

**4-Methylphenyl-2,2,2-trifluoroethyl diethylamidophosphate 3b is as follows**: It is a colorless liquid, yielding 78%, E_b0.01mmHg_ = 100°C. ^1^H NMR *δ*: 1.0 (t, 3H, CH_3_); 2.3 (s, 3H, CH_3_-Ph); 3.2 (m, 4H, CH_2_N); 4.32 (m, 2H, OCH_2_-); 7.1 (m, -Ph-, 4H). ^31^P NMR *δ*: 5,53 (11 peaks, ^3^J_P-H_ = 7.3Hz). ^19^F NMR*δ*: −75,4 (t, ^3^J_H-F_ = 8,5 Hz).^13^C NMR*δ*: 13.8 (CH_3_CH_2_); 20.7 (CH_3_Ph); 39.9 (2NCH_2_CH_3_); 62.7 (q, CH_2_CF_3_; ^3^J = 33 Hz); 122 (q, CF_3_, ^1^J = 267 Hz); (117; 130; 134; 148; C_arom_). ESI MS m/z 348 [M+Na]^+^; 673 [2M + Na^+^]; C_13_H_19_F_3_NO_3_P: calc. 325.1055; found 325.1056

**4-Methylphenyl-2,2,2-trifluoroethylpiperidin-1-ylphosphonate 3c is as follows: **It is a colorless liquid, yielding 95%, NMR *δ*: ^31^P**:** 3.95 (7 raies, ^3^J_P-H_ = 7.9 Hz); ^19^F**: −**75.4 (t, ^3^J_H-F_ = 8.5 Hz); ^13^C**:** 19.6 (CH_3_Ph); 23.2 (CH_2_); 24.7 (2CH_2_); 44.5 (2CH_2_N); 62.7 (q, CH_2_CF_3_; ^2^J =37 Hz); 122 (q, CF_3_, ^1^J = 276 Hz) (117; 130; 134; 148; C_arom_); ^1^H: 2.3 (s, 3H, CH_3_-Ph); 7.1 (m, -Ph-, 4H).

**4-Methylphenyl-2,2,2-trifluoroethylmorpholin-4-ylphosphonate 3d is as follows: **It is a colorless viscous liquid, with yield 95%, NMR *δ ***: **^31^P**:** 2.92 (7 raies, ^3^J_P-H_ = 7.3 Hz); ^19^F: −75.4 (t, ^3^J_H-F_ = 7.3 Hz); ^13^C**:** 20.5 (CH_3_Ph); 44.5 (2CH_2_N); 62.9 (q, CH_2_CF_3_; ^2^J = 33 Hz); 66.6 (2OCH_2_); 122.0 (q, CF_3_, ^1^J = 267 Hz) (119; 130; 134; 148; H_arom_); ^1^H: 2.3 (s, 3H, CH_3_-Ph); 3.2 (m, 4H, CH_2_N); 3.6 (m, 4H, OCH_2_); 4.32 (m, 2H, OCH_2_CF_3_); 7.1 (m, -Ph-, 4H).

#### 2.1.4. Synthesis of 2,2,2-trifluoroethyl(4-nitrophenyl)phosphonochloridate

A solution of trifluoroethanol (18 mmol) and triethylamine (18 mmol) in 20 mL was added at 0°C to a solution of p-O_2_NPhP(O)Cl_2_ (15 mmol) in 120 mL of anhydrous ether under a flew of nitrogen. After 48 h of stirring at room temperature, the precipitate is filtered and the filtrate is concentrated and then distilled. It is a yellow viscous liquid, with yield = 61%, E_b0.5mmHg_ = 138°C, NMR *δ ***: **^31^P**: −**0.43 (t, J = 9.7 Hz); ^19^F**: −**75.0 (t, J = 8.5 Hz); ^13^C**:** 65. 0 (q, OCH_2_CF_3_, J = 32 Hz); 122 (q, CF_3_, J = 274 Hz); (154, 146, 127, 121 C_arom_); ^1^H**:** 8.3 et 7.4 (2m, 4H_arom_); 4.6 (m, 2H, OCH_2_CF_3_).

#### 2.1.5. Synthesis of 4-nitrophenyl-2,2,2-trifluoroethylphosphoramidates

6 mmol of 2,2,2-trifluoroethyl (4-nitrophenyl) phosphonochloridate in 50 mL of anhydrous ether was added to 13.2 mmol of triethylamine in 10 mL of anhydrous ether under nitrogen. Stirring is continued for 5 hours. The precipitate is filtered and the filtrate was concentrated to give an oil, which unless otherwise is not further purified.

**2,2,2-Trifluoroethyl-4-nitrophenyl-*N*,*N*-dimethylphosphoramidate 6a is as follows: **It is colorless viscous liquid, with yield (94%), NMR *δ ***: **^31^P**:** 4,85; ^13^C**:** 36,6 (CH_3_N); 63,5 (q, CH_2_CF_3_, J = 33 Hz); 122 (q, CF_3_, J =275 Hz); (164, 157,156, 145, 126, 121, C_arom_); ^1^H**:** 2,8 (d, 2CH_3_); 4,4 (m, CH_2_); 7.4 et 8.2 (2m, 4H_arom_).

**2,2,2-Trifluoroethyl-4-nitrophenyl-*N*,*N*-diethylphosphoramidate 6b is as follows: **It is a yellow viscous liquid, with yield (58%), E_b0.01mmHg_ = 152°C, NMR *δ*: ^31^P**:** 5.0 (hept. J = 7.4 Hz); ^13^C: 36.6 (CH_3_N); 63.5 (q, CH_2_CF_3_, J = 33 Hz); 122 (q, CF_3_, J = 275 Hz); (154, 146, 126, 121, C_arom_); ^19^F:** −**73.6 (t, J = 8.5 Hz), ^1^H**:** 1.0 (t, CH_3_); 3.2 (m, CH_2_CH_3_); 4.4 (m, CH_2_); 7.4 et 8.2 (2m, 4H_arom_).

**2,2,2-Trifluoroethylmorpholin-4-yl(4-nitrophenyl)phosphoramidate 6c is as follows: **It is a yellow solid, with yield (95%), RMN *δ*: ^31^P**:** 2.4 (hept. J = 7.9 Hz); ^13^C**:** 45.0 (2CH_2_N); 62.7 (q, CH_2_CF_3_; ^2^J = 35 Hz); 67,0 (2OCH_2_); 122 (q, CF_3_, J = 275 Hz); (163, 156, 144, 126, 121, C_arom_),^19^F:** −**75.6 (t, J = 8.5 Hz); ^1^H**:** 3,1 (m, 4H, CH_2_N); 3.6 (m, 4H, OCH_2_); 4.32 (m, 2H, OCH_2_CF_3_); 7.4 et8.2 (2m, 4H_arom_).

### 2.2. Biological Activity

#### 2.2.1. Antimicrobial Activity

Different bacterial strains are maintained by subculture on BHI agar (Brain Heart Infusion, agar and brain-heart infusion) favorable to their growth for 24 hours* C. B. cereus *at 37°C with the exception of* L. monocytogenes* and incubated at a temperature of 30° grown on nutrient agar at 30°C. The agar diffusion method (method of disc). Filter paper disc was impregnated by different tested compounds and deposited on the surface of agar petri dishes. Minimal inhibitory concentrations were determined by the dilution method in solid medium.

#### 2.2.2. Anticholinesterase Activity

Chemicals : Acetylcholinesterase (AChE) type VI-S, from electric eel 137 U/mg solid, 217 U/mg protein, 5,5′-dithiobis[2-nitrobenzoic acid] (DTNB), acetylthiocholine iodide (AChI), tris[hydroxymethyl] aminomethane (tris buffer), and dimethylsulfoxide (DMSO) were supplied from Sigma-Aldrich. Acetylcholinesterase enzymatic activity was measured by the Ellman test [21]: 98 *μ*L (50mM/L) tris-HCl buffer (pH 8), 30 *μ*L of the sample, and 7.5 *μ*L of the acetylcholinesterase solution containing 0.26 U/mL were well mixed in 96-well microplates and incubated for 15 min. Subsequently, 22 *μ*L of (3mmol/L) DTNB was added. The absorbance at 405 nm was read when the reaction reached the equilibrium. A control reaction using DMSO instead the sample and a blank with Tris-HCl buffer instead of enzyme solution were used. Tests were carried out in duplicate.

Inhibition, in %, was calculated in the following way: I (%) = 100 - (A sample/A control)*∗*100,

where A sample is the absorbance of the sample containing reaction and A control the absorbance of the reaction control.

## 3. Results and Discussion

### 3.1. Synthesis

**Figure 1:**
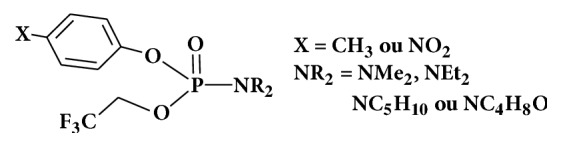
Designed phosphoramidates.

The design of arylphosphoramidates in this work (Figure 1) is based on phosphoramidate structures already used as prodrugs. (NR_2_) is the masking group which hydrolyzes first. (Ar-X) is the leaving group and (OR') is the active group that should be supplied to the cell to be treated, avoiding hydrolysis thereof to the surface of the cell by the NR_2_ group in Figure 1.

**Scheme 1:**
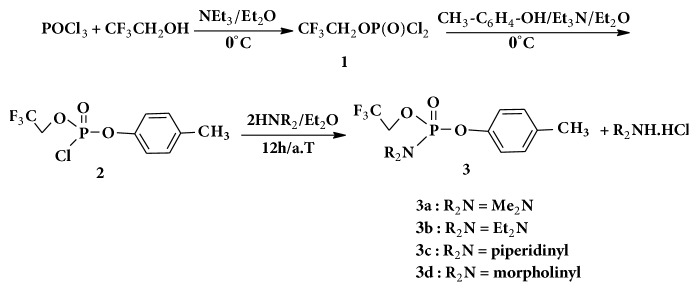
Synthesis of p-tolylphosphoramidates** 3**.

For the synthesis of the designed arylphosphoramidates, several attempts have been carried out. On the basis of the reported literature by [2], we have attempted the synthesis of the phosphoramidates (R_2_N)P(O)(OCH_2_CF_3_)(OphCH_3_) in a one pot by mixing phosphorus oxychloride, alcohols (HOPhCH_3_, HOCH_2_CF_3_), and the amine (R_2_NH) as shown in Scheme 1. However, in addition to the expected phosphoramidates, the ^31^P NMR spectrum showed signals relating to the formation of several byproducts such as CF_3_CH_2_OP(O)Cl, (CF_3_CH_2_O)_2_P(O)(OPhCH_3_), and P(O)(OPhCH_3_)_3_. These byproducts could not be separated by distillation. The reaction was then undertaken in multisteps with several assays: first, the p-cresol and triethylamine are added to phosphorus oxychloride in anhydrous ether at −10°C and kept at room temperature for 12 hours. The corresponding ^31^P NMR spectrum showed the corresponding dichlorophosphate in addition to other unknown phosphorus compounds. Then, the addition of alkylamine on phosphorus oxychloride followed by the addition of HOCH_2_CF_3_ gave the desired dialkylphosphoramidic dichloride. However the addition of CF_3_CH_2_OH in the presence of DMAP as catalyst afforded the expected phosphoramidate in low proportion with the appearance of a new compound due to the substitution of the-NR_2_ group by –OR group located at -6 ppm. Finally, we have reacted POCl_3_ with CF_3_CH_2_OH in presence of triethylamine in anhydrous ether for 12 hours at room temperature and subsequent addition of p-cresol and amine afforded the desired compounds** 3** with good yields and satisfactory purity. In these optimized conditions, the other amines were used and gave the corresponding phosphoramidates (Scheme 1).

**Figure 2:**
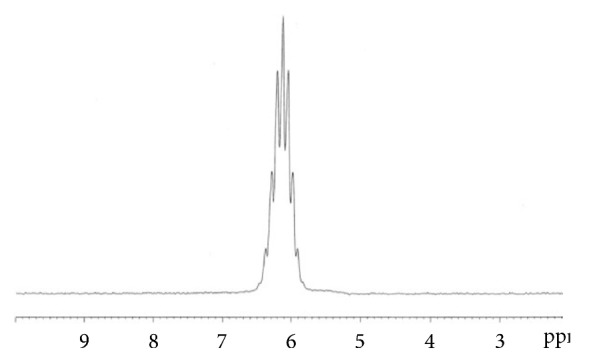
^31^P NMR of** 3a** in CDCl_3_ at 298 K.

The ^31^P NMR coupled to ^1^H spectrum of the compound** 2** showed a triplet at 0.5 ppm with a coupling constant value ^3^J_H-P_ = 8 Hz with the two protons of the methylene group. The reaction of two equivalents of amine in anhydrous ether with compound** 2** for 12 hours at room temperature gave the pure arylphosphoramidates** 3**. The ^1^H NMR spectrum of compound** 3a** shows a doublet at 2.8 ppm resulting from the coupling with the phosphorus atom. The methylene group shows a multiplet at 4.4 ppm due to the coupling with both fluorine and phosphorus atoms. The corresponding ^31^P NMR spectrum (Figure 1) shows a multiplet of 9 peaks resulting from the coupling between the phosphorus atom and 8 protons (CH_2_O and 2CH_3_). ^19^F NMR spectrum shows a triplet due to coupling of the fluorine atom with the methylene group (Figure 2).

**Scheme 2:**
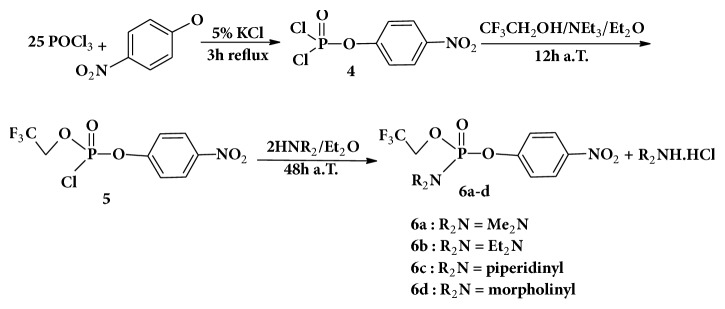
Synthesis of p-nitrophenol phosphoramidates** 6**.

We have also used nitrophenol instead of p-cresol following the same sequence described for the synthesis of compounds** 3**. However the purification of the reaction products was tedious and gave a mixture of products together with the desired phosphoramidates. We have therefore attempted to do the synthesis using a different sequence where the addition of the amine with phosphorus oxychloride was followed by nitrophenol and then by trifluoroethanol allowing the desired phosphoramidate but the reaction took 5 days. Finally using the starting compound** 4** described in the literature [20], the reaction with trifluoroethanol gave the corresponding chlorophosphoramidate in a good yield. The reaction of the compound** 5** with two equivalents of amine in anhydrous ether at room temperature for 48 hours led to the desired phosphoramidates with yields ranging from 58 to 95% (Scheme 2).

**Figure 3:**
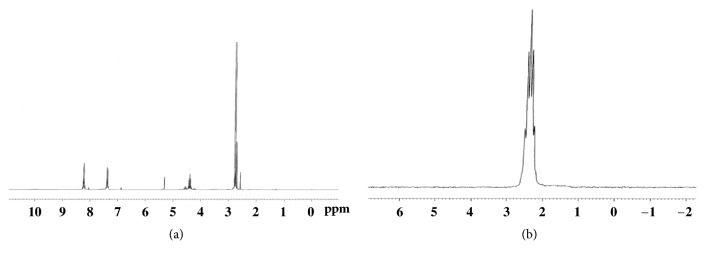
(a) ^1^H NMR of** 6a** in CDCl_3_ and (b) ^31^P NMR coupled to ^1^H of** 6d** in CDCl_3_.

The ^1^H NMR spectrum of the compound** 6a** shows the coupling with methyl protons with the phosphorus atom at 2.8 ppm (Figure 3). On the other hand, the ^31^P NMR spectrum of** 6d** shows a multiplet of seven peaks reflecting the coupling between phosphorus atom and methylene protons at 2.4 ppm, at lower field compared to p-tolylphosphoramidate. The spectroscopic data of these arylphosphoramidates are shown in Table 1.

The above results in Schemes 1 and 2 show that the formation of phosphoramidates is sensitive to the nature of the alcohols used. Thus preparing phosphoramidates**3 **is in the order CF_3_CH_2_OH followed by p-cresol and the amine, whilst for phosphoramidates** 6**, the addition of the aromatic alcohol, nitrophenol, should be the first step then the addition of the second alcohol CF_3_CH_2_OH and amine as the final step.

### 3.2. Biological Activity

#### 3.2.1. Antimicrobial Activity

**Figure 4:**
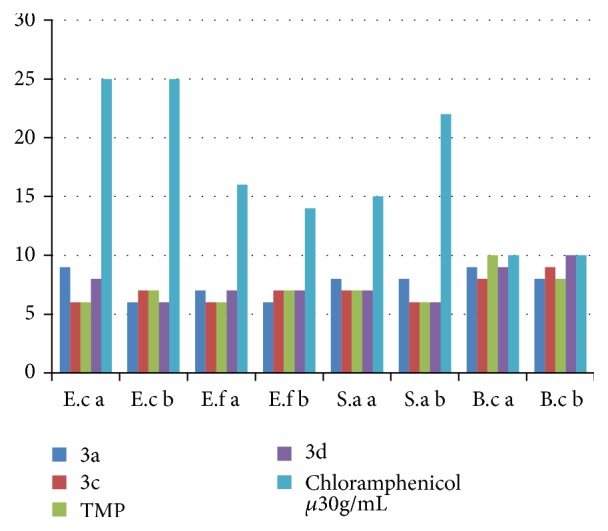
Inhibition diameters zones of bacterial growth (mm) of phosphoramidates** 3a, c, d, **and** TMP**.* E.c a: Escherichia coli* ATCC 8739; E.c b:* Escherichia coli* DH5*α*; E.f a:* Enterococcus faecalis* ATCC 29212; E.f b:* Enterococcus faecium* ATCC19436; S.a a:* Staphylococcus aureus* PIC 4.83; S.a a:* Staphylococcus aureus* ATCC 25923; B.c 49:* Bacillus cereus* 49; B.c:* Bacillus circulans* (ATCC: American Type Culture Collection; PIC: Pasteur Institute Collection).

The phosphoramidates** 3a**,** 3c, **and** 3d** have been tested towards different Gram negative and Gram positive bacteria. Chloramphenicol was taken as reference to study the effect of different substituents on biological activity. The compound 2,2,2-trifluoroethyl N,N,N′,N′-tetramethylphosphorodiamidate (**TMP**) [22] has also been tested in order to evaluate the effect of electrodonating effect on the phosphorus atom. The reactivity of each compound was evaluated towards the different bacterial strains by the agar diffusion method. The inhibition diameters of bacterial growth area are summarized in Figure 4.

The results show that all the tested compounds in a pure state have diameters of inhibition zone of bacterial growth ranging between 6 and 10 mm for all strains of Gram negative and Gram positive bacteria. The compounds** 3a**,** 3c, 3d,** and** TMP **do not exhibit a particular antimicrobial activity. To better assess the sensitivity of the strains towards the activity of these compounds, their minimum inhibitory concentration (MIC) was determined by the dilution method on solid medium. The results show that these values (i.e., 1000 to 2000 *μ*g/mL) are high compared to those of usual therapeutic agents. The lack of antimicrobial activity may be due to the low solubility in water [23, 24], as well as instability in alkaline hydrolysis [25].

#### 3.2.2. Antiacetylcholinesterase Activity

**Figure 5:**
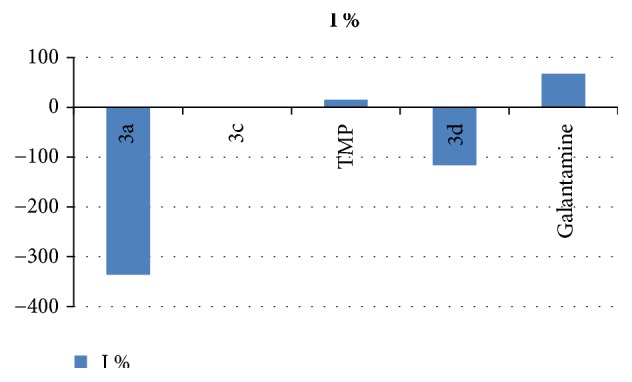
Measurements of optical density DO406.

The determination of the inhibitor activity of acetylcholinesterase (AChE) of compounds** 3a, c, d, **and** TMP** with galantamine, taken as a reference, was performed according to the method of Ellman [21]. The results of optical density measurements of all the tested compounds are shown in Figure 5.

As can be seen from Figure 3, the negative values of the phosphoramidates** 3a** and** 3d **indicate that the compounds have no inhibitory activity against AChE. The compounds** 3c **and** TMP **exhibit some AChE activity. The compound** 3c** is more active than the compound** 3d **probably due to the hydrophobicity and the more electrodonating character of** 3d.** However, the difference in activity found between phosphoramidates** 3a** and** 3c** both bearing electrodonating groups could be mainly due to steric hindrance which would enhance AChE activity in** 3c**. On the other hand, the direct substitution of phosphorus atom by amine group in** TMP **can enhance sensitively of the AChE inhibitory effect. This electrodonating group enhances nucleophilic character which facilitates the nucleophilic attack on the phosphorous atom and the elimination of the leaving group. This is consistent with the literature [26, 27] which showed that the AChE inhibition increases when the polarity of the amine group increases related to the electrostatic attraction between this group and the enzyme which becomes stronger.

Therefore the inhibitor-enzyme interaction would be influenced mainly by the reactivity of the phosphorus atom, which determines the rate of the phosphorylation reaction and the ease of bonding between the inhibitor and the enzyme to form a complex before the phosphorylation step and the electronic and steric effects of hydrophobic moieties directly bounded to the phosphorus atom. The binding affinity is determined by the structural features in particular the instability of the P=O bond as reported in the literature [28, 29] which may also influence the cholinesterase activity.

## 4. Conclusions

In this paper, we have synthesized new phosphoramidates R_2_N(pX-ArO)P(O)OR′ using convenient steps. All synthesized phosphoramidates were characterized by ^31^P NMR, ^1^H, and ^13^C NMR, IR spectroscopy. The biological study of some of arylphosphoramidates did not show particular antibacterial activity even when the phosphorus atom was directly substituted by an electrodonating group (-N(Me)_2_). However the AChE activity has shown that the directly substituted electrodonating group on the phosphorus atom has some AChE inhibitory effect. Therefore the substituents nondirectly bounded to the phosphorus atom did not affect sensitively the reactive sites of the arylphosphoramidates towards AChE enzyme.

## Figures and Tables

**Table 1:** Spectroscopic data of arylphosphoramidates *δ*^31^P (ppm),^3^J (Hz), and *ν*_PO_ (cm^−1^).

**Phosphoramidates**	**yield**%	**δ** ^**31**^ **P (ppm) **	^**3**^ **J (Hz)**	***ν***_**PO**_ ** (cm** ^**-1**^ **)**
**3a**	91	6.15	9,7	1167
**3b**	80	5.53	7,3	1165
**3c**	95	3.95	7,9	1165
**3d**	90	2.92	7,3	1168
**6a**	60	4.85	9,7	1180
**6b**	58	5.0	7,4	1181
**6c**	90	3.1	7,5	1180
**6d**	95	2.4	7,9	1178

## Data Availability

The data used to support the findings of this study are available from the corresponding author upon request.
